# Evaluating Effectiveness of Outpatient Monitoring in Type 2 Diabetes: The One-Year Experience in an Italian Group of Primary Care

**DOI:** 10.3390/ijerph182111540

**Published:** 2021-11-03

**Authors:** Francesca Lazzarini, Luca Barbacane, Giuseppe Scoleri, Rosanna I. Comoretto, Gianni Cogno, Benedetta Disarò, Luigi Gomirato, Francesca Stocco, Alessandro Suppa, Gianluca Toninato, Clara Minto, Danila Azzolina, Sabino Iliceto, Dario Gregori

**Affiliations:** 1 Department of Cardiac, Thoracic, Vascular Sciences and Public Health, University of Padua, 35131 Padua, Italy; francesca.lazzarini@live.com (F.L.); rosanna.comoretto@unipd.it (R.I.C.); clara.minto@ubep.unipd.it (C.M.); danila.azzolina@unipd.it (D.A.); sabino.iliceto@unipd.it (S.I.); 2 Group of Primary Care of Martellago, Unità Locale Socio-Sanitaria ULSS 3 Serenissima, 30030 Martellago, Italy; luca.barbacane@aulss3.veneto.it (L.B.); pinoscoleri@libero.it (G.S.); dr.giannicogno@gmail.com (G.C.); disaro.benedetta@gmail.com (B.D.); luigigomirato@alice.it (L.G.); stocco.francesca.m@gmail.com (F.S.); suppa_alex@libero.it (A.S.); gitoni61@yahoo.it (G.T.)

**Keywords:** type 2 diabetes mellitus, primary care, care plan, effectiveness, chronic disease management, longitudinal analysis

## Abstract

Nowadays, chronic disease management is the primary challenge of the healthcare system. From 2015, in the Veneto region (Italy), patients with a diagnosis of type 2 diabetes mellitus (T2DM) have been included in the diagnostic-therapeutic pathway (PDTA) program, and their clinical condition has been continuously monitored. The aim of this retrospective study is to determine the effectiveness of PDTA intervention, alone or in combination with a specialized one, in subjects with diagnosis of T2DM. Clinical and behavioral characteristics were collected at baseline and after 1 year of follow-up. Two subgroups were considered: subjects enrolled in PDTA only and subjects enrolled in both the PDTA program and in the care plan proposed by the specialized medical center (CAD group). Longitudinal analysis showed a relevant positive effect of time on diastolic blood pressure, while CAD enrollment appears to be related to higher levels of glycated hemoglobin. When included together in the same model, interaction between time and CAD covariates results in completely nonsignificant effects. As long-term management of chronic disorders, such as T2DM, is often difficult due to disease characteristics and problems in healthcare organization, monitoring programs, such as PDTA, and specialized care programs, such as CAD, do not show a clinically relevant effect in the first year of follow-up. Therefore, they should be analyzed over a longer period. However, they should also carefully consider the need for adequate tools for data collection and sharing, in addition to the context of application, patient expectations and the need for a long-term educational program.

## 1. Introduction

Chronic disease management is one of the primary challenges of the healthcare system. The current economic crisis scenario, the lack of resources and changes in patient needs prompted healthcare providers to imagine new care strategies. Among several organizational approaches, the common denominator seems to be the concept of interoperability: different systems being able to mutually interface using the same language to achieve the same result. The Chronic Care Model can be considered the medical application of the concept of interoperability in informatics, as it aims to implement a link among individual clinical conditions, available resources in the community and resources offered by healthcare systems [1]. This is also the fundamental paradigm of the diagnostic-therapeutic pathway program, called PDTA (percorso diagnostico terapeutico assistenziale), a management strategy adopted by the Italian region of Veneto to support and implement care activities for patients with chronic disease. Type 2 diabetes mellitus (T2DM) is one of the first diseases in which the PDTA strategy has been applied in the Veneto region.

Diabetes is one of the main chronic diseases that affects the world’s population. In 2015, the International Diabetes Federation estimated that around 415 million people worldwide were affected by this disease [2]. In Italy, diabetes affects more than 3.5 million people (5.5% of the total population), with around 250,000 new cases each year [2,3]. As diabetes is a chronic degenerative disease associated with a high risk of chronic complications and comorbidities, costs related to this pathology have a great impact on total expenditure of healthcare systems, and in Italy an economic burden of €20.3 billion/year has been estimated [4].

In the Veneto region, the prevalence of diabetes is around 5.3% [5], and these patients can be treated at the centralized level (in-hospital care) or at decentralized level (outpatient territorial care), depending on the stability of clinical conditions and the needs of patients. The clinical pathway starts with the diagnosis of T2DM or with the identification of conditions that can predispose the subject to metabolic disease. After diagnosis, patients could be involved in periodic clinical examinations performed by a territorial specialized medical center for diabetes treatment (CAD, Centro Anti-Diabetico). Therefore, the clinical pathway of patients with T2DM takes place mainly outside of the hospital, in a decentralized healthcare setting. Recently, following the PDTA strategy, subjects with a diagnosis of T2DM are involved in a long-term surveillance program conducted by family physicians, which substitutes or integrates the clinical activity of CAD. This new care program has been created to improve the management of patients with T2DM, and it is based on periodic medical examinations performed by family physicians and specialized nurses.

The main aims of the PDTA program are the integration of activities and interventions between hospital and territory, the identification of the best care path in terms of appropriateness according to a process point-of-view and the maintenance of a balance avoiding situations of decompensation or the onset of long-term complications. It also uses specific indicators to ensure continuous improvement of interventions. On the other hand, CAD is a center managed by diabetes specialists. Its care program is aimed at short- and medium-term outcomes, including the control of drug therapy, biochemical tests and the onset of complications. Therefore, the substantial difference between the two approaches is that the first is more like a monitoring approach, while the second is one of specialized care.

This study aims to evaluate the effects of the PDTA strategy, alone or in combination with CAD care, on the main biochemical parameters related to hyperglycemia, anthropometric measures and lifestyle of patients affected by T2DM.

## 2. Materials and Methods

### 2.1. Study Design and Setting

This retrospective observational study was conducted in the primary care setting of the Venice Local Health Unit (ULSS 3 Serenissima). The integrated primary care network, located in Martellago, includes eight family doctors and four specialized nurses that offer healthcare to outpatients for routine check-up. Subjects who referred to this network were about 11,674 patients with chronic diseases and comorbidities requiring long-term assistance, of whom 840 had a diagnosis of T2DM. Both family doctors and nurses are involved in the organization and management of patients’ care plan, pharmacological treatment, specialist examinations, self-care education and disease surveillance. All subjects referred to the primary care network with a diagnosis of T2DM and at least two examinations included in the PDTA program carried out in the period between November 2015 and May 2017, were evaluated for study enrollment. Criteria for eligibility were more than 18 years of age and absence of cognitive impairment or severe comorbidities (such as the final stage of cancer, liver disease or renal failure). The follow-up time was defined as the number of days between the first and second examinations. All involved patients expressed their informed consent after explaining the objectives and design of the study. The study design and data collection were carried out in full respect of the regulation of clinical practice (Italian Decree Law 211/2003) and current legislation on the treatment of personal information (Italian Legislative Decree 196/2003).

### 2.2. Data Collection

**Figure 1:**
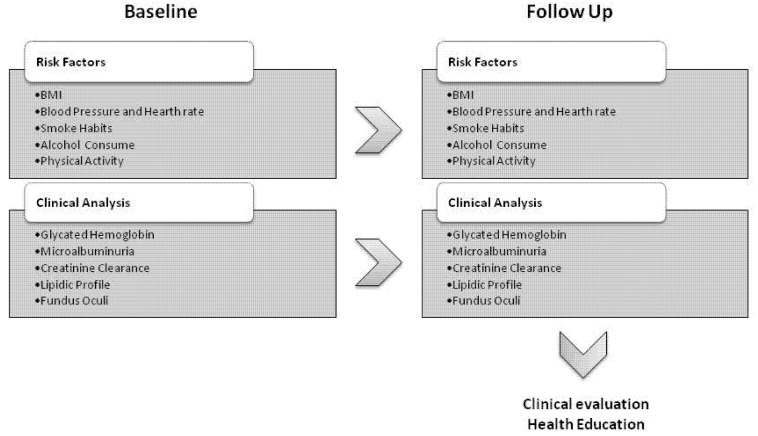
PDTA organization and data collected during baseline and follow-up examinations. Abbreviations: BMI, body mass index.

The collection of relevant information was carried out by a specialized nurse, through the manual extraction from historical medical registries. In the primary care network, patient data were recorded using an online platform that allows physicians and nurses to share information and give periodic feedback to the regional diseases surveillance system. The collected data included general information (date of birth, age and sex), details on the pharmacological treatment for diabetes and data obtained from the two PDTA examinations. Baseline and follow-up data collected within the PDTA program included results of biochemical analysis (glycated hemoglobin, microalbuminuria, creatinine clearance, lipid profile) and the evaluation of weight, height, body mass index (BMI), waist circumference, smoking habits, alcohol consumption, physical activity, systolic and diastolic blood pressure and heart rate. Details on the PDTA organization and collected data are reported in Figure 1.

### 2.3. Statistical Analysis

Descriptive statistics of the sample are reported using median and interquartile range (I–III quartile) for continuous variables and absolute number and percentage for categorical ones. We divided the study sample into two subgroups: subjects who were involved in the PDTA program only (the PDTA group), and subjects who were involved in both the PDTA program and the CAD care plan (the PDTA and CAD group). The statistical significance of the differences between the two groups was evaluated using Wilcoxon–Kruskal–Wallis for continuous variables and Pearson’s chi-square test for categorical ones. A mixed-effects model for longitudinal analysis was applied to evaluate changes in clinical and biochemical variables from baseline to follow-up. Assuming that all subjects involved were followed with a PDTA approach, we first conducted a longitudinal analysis considering just the time effect (Model 1), and a second longitudinal analysis including both time and CAD care plan involvement as covariates (Model 2). In the last model, we evaluated the effects of three different factors: (i) time; (ii) CAD care plan involvement; (iii) interaction of both time and CAD involvement. For each longitudinal analysis, the *p*-values were adjusted by multiplicity using the test proposed by Benjamini and Hochberg [6].

### 2.4. Sample Size

Sample size estimation was conducted using a Monte Carlo (MC) simulation procedure.

Five hundred replications were considered. For each MC repetition, the hemoglobin glycated values were generated considering this outcome modelization:

Y = 51 + 2.6 ∗ Time + 2.6 ∗ CAD + 2.6 ∗ Time ∗ CAD(1)

These assumptions were considered:

- The time, CAD and interaction effects were all hypothesized to be equal to 2.6. The effect of 2.6 corresponds to a Cohen d effect size of 0.8 [7] (d = (51 − 53.6)/3.3 = 2.6/3.3 = 0.8);
- Both marginal and interaction terms were considered in the data generation process;
- The baseline glycated hemoglobin value was supposed to be equal to 51 [8];
- The outcome variance was assumed to be equal to 3.3 [8].

The CAD and the “time-CAD” interaction effects on glycated hemoglobin were estimated, for each simulation, using the Generalized Linear Mixed Model (GLMM) that accounts for a random intercept term for two repeated measures.

A sample size of 124 subjects ensured a significant effect of both the CAD and “CAD-time” interaction terms among 87% of MC replications. The alpha level was adjusted for multiplicity among 17 outcomes (alpha = 0.05/17).

Analyses were performed using the R System, the RMS libraries [9,10] and the simstudy packages [11].

## 3. Results

The study population included 124 patients with a diagnosis of T2DM, 91 of them were included in only the PDTA program and 33 were involved in both the PDTA and the CAD pathways (Figure S1, based on the STROBE statement [12]). The median age was 71.5 years, and the study population was mainly composed of male subjects (61%). Most patients were treated with oral hypoglycemic agents (90%), while only a few subjects received insulin therapy (3%) or no treatment (7%). The median follow-up time between baseline and the second PDTA examination was estimated to be 370 days. General information on sex, age and pharmacological treatment of the two population groups is reported in Table 1.

Table 2 and Table 3 include the baseline data and the data collected during the second PDTA examination, respectively. At baseline, patients in the PDTA and CAD group appeared to have worse general conditions than those in the PDTA group: they had higher values of glycated hemoglobin and triglycerides, and lower level of high-density lipoprotein. Baseline anthropometric variables, such as BMI and waist circumference, did not differ between groups, nor did smoking and alcohol consumption. Most of the patients reported no physical activity or a moderate level of exercise. Data from follow-up examination confirmed a difference in the glycated hemoglobin value between the two groups, while the lipid profile seemed to be similar regardless of CAD intervention. Moreover, after one year, microalbuminuria showed higher values in the PDTA group.

The results of the longitudinal analyses are reported in Table 4. Model 1 did not consider the potential confounding effects of the involvement in the CAD care plan. In this case, the estimated change from baseline to follow-up was positive just for diastolic blood pressure (DBP) and alcohol consumption. By adding the involvement in the CAD care-plan as a covariate (Model 2), the analysis of the time effect confirmed the only statistically significant change in DBP. The effect of CAD enrollment led to an increase in glycated hemoglobin, while the interaction between CAD involvement and time resulted in completely nonsignificant effects.

## 4. Discussion

To the best of our knowledge, this is the first study aimed at verifying the effectiveness of the PDTA intervention, alone or in combination with the CAD program, in patients with T2DM after 2 years of its implementation, by evaluating changes in the biochemical variables of the patients. It is necessary to emphasize that the results obtained, although significant from a statistical point of view, cannot be considered all clinically relevant. In fact, the effects reported by the models are very small from a clinical point of view. Our findings demonstrated a substantial lack of any statistically significant variation (and therefore a lack of any clinically relevant change) in all clinical parameters between baseline and approximately 1 year of follow-up. The two models in the longitudinal analysis have been used to investigate the underlying differences between the PDTA approach used alone and its combination with the CAD one. As all subjects were followed with the PDTA program, the first model reported the effect of the follow-up time on the PDTA program. In the second model, both follow-up time and the CAD approach were together analyzed. Therefore, results of the second model considered the adjusted effect of both covariates: it indicated whether there are ameliorative effects on patients’ conditions if they were followed with both approaches. Looking at models’ results, the integration of the two approaches (Model 2) did not seem to lead to any improvement in clinical parameters compared to the application of PDTA alone (Model 1). However, it is important to note that the effect of the CAD approach in Model 2 is associated with an increase in glycated hemoglobin, and this effect, although clinically small, could be noteworthy in terms of a general pattern: a decrease in this parameter would be expected, while its increase was observed. Furthermore, patients followed according to only the PDTA criteria did not show any improvement in clinical parameters.

To explain these results and compare this PDTA program with previous similar experiences, we identified three main issues that can partially explain these findings: (i) the low applicability of the program, (ii) the poor relevance of the indicators of T2DM and (iii) the short follow-up time chosen in the present study.

The applicability (and success) of a healthcare program is determined mainly by its ability to comply with characteristics of the setting and population of interest; the possibility to offer easy tools with a high grade of usability, not requiring much effort by the healthcare providers; its economic sustainability for a long time period; its consistency with international guidelines and scientific evidence on the specific disease; and the statement of clear and shared objectives that should be perceived as fundamentals by health professionals directly involved in the program realization.

An in-depth examination of the PDTA structure highlighted some important issues related to compliance with the application setting. In fact, the territorial and decentralized healthcare systems in the Veneto region did not have a unique informative database: each clinic, especially at the decentralized level, can also choose a different online application to collect and manage data for a common PDTA program. This led to a wide fragmentation of the information. Moreover, the profiles of patients with T2DM are extremely complex: theoretically, this information should include data collected from all specialists involved in the clinical pathway, such as family physicians and diabetologists, but also from every other care provider that actively contributes to the patient’s assistance. During the realization of the present study, we faced a substantial lack of informative integration between the family physicians care plan (PDTA) and the one carried out by the specialized center for diabetic treatment (CAD). Both clinical structures provided medical examinations and similar information collected without any mutual sharing. Family physicians were only informed about the subject’s involvement in the CAD program and about results of biochemical analysis, if any. All other parameters, such as physical activity or eating habits, as well as the content of the information given to the patient about self-care and everyday disease management were not explained. In the same way, clinical records of subjects enrolled in PDTA were only collected by family physicians. The presence of different online platforms probably generated several technical difficulties in data sharing between clinical structures. In a 2011 statement, the European Commission declared the urgent need for a large-scale national system capable of ensuring data sharing among healthcare providers, suggesting to all member countries the adoption of specific strategies [13]. Following this and other recommendations, worldwide healthcare systems focused on the implementation of Electronic Health Records (EHRs) as key elements to improve informational continuity of care [14]. Today, the situation in Italy and also in the Veneto region is still inadequate for what concerns the use of shared informative databases, in particular in territorial settings.

Moreover, the reason for the current ineffectiveness of PDTA can be ascribed not only to technical and organizational issues but also to the nature and structure of the intervention itself. Uncertain aspects of the PDTA program for T2DM may include the requirement of only one medical examination during a year, the absence of a well-organized health education program and the difficulties in working integration between nurses and family doctors within the primary care clinic. In our study, the implementation of the PDTA program was difficult due to resource management and personnel deficiency. Data collection was completed mainly by nurses, but high workload often impeded a complete and in-depth examination able to detect the health needs of the patient.

The second issue is related to the relevance of the T2DM indicators, and refers to the ability to collect clinical variables to provide a reliable picture of the pathways of patients. PDTA indicators include a wide range of anthropometric parameters, everyday habits and biochemical variables. However, some other details could be included to provide a more complete analysis of the clinical history of the individuals: the number of hospital admissions related to T2DM, the development of comorbidities, dietary habits, pharmacological treatments and adherence to medical prescriptions are often used to assess the effectiveness of community-based programs [15,16]. However, also in this case, the lack of interoperability among different informative systems entails considerable difficulty in overcoming this issue.

The third aspect that may have influenced the observed results is that the severity of the disease was not considered as a confounder. As reported in the literature, the severity of the disease or its clinical stage can have an important influence on the results of health (both prevention and treatment) programs [17]. However, since the study population was already very small due to inclusion criteria, the addition of this stratification would have led to further fragmentation of the results. Finally, the shortness of the follow-up chosen for the present study may be a potential limitation in the evaluation of PDTA effectiveness. Our study was carried out through the analysis of patients who underwent at least two medical examinations included in the PDTA program. Therefore, further research should consider increasing both the follow-up time and the study cohort in order to capture significant changes in clinical parameters, also introducing clinically relevant stratifications.

Management strategies in the field of healthcare should consider the specific characteristics of chronic disease, the subject’s expectations, and the life context. Patients with T2DM often do not perceive their condition to be serious, and do not realize the high probability of severe long-term consequences. In individuals with type 2 diabetes, nonadherent behavior to medical prescription has been estimated to range from 50% to 93% [18]. A recent study has identified three main triggers influencing adaptation to the disease: individual context (negative/positive beliefs about the illness, personal background), supportive system (family, community, human interactions in health organizations) and self-comparison (comparison of diabetes with other diseases or comparison with other diabetic subjects) [19]. For diabetics, diet and physical activity are the main—and most difficult—lifestyle changes. Some studies suggest that possible reasons for nonadherence to physical exercise could be the lack of information, the perception that exercise exacerbates the disease, the absence of a supportive partner, a negative family history of diabetes mellitus and lower socioeconomic class. Moreover, dietary habits could be negatively influenced by older age, poor knowledge about diabetes mellitus, poor self-discipline and the tendency to eat outside of the home [20,21]. Promotion of physical activity, weight maintenance and healthy nutrition should be improved to reduce both the incidence and worsening of T2DM and to guarantee long-term adherence to medical indications. Previous studies showed how diagnosis acceptance and self-management skills could be improved after a group educational intervention [22,23].

## 5. Conclusions

Long-term management of chronic disorders, such as T2DM, is often difficult due to the characteristics of the disease and the problems of the healthcare organization. Both monitoring programs, such as the novel PDTA, and specialized approaches, such as CAD care, in a limited time period (1 year of follow-up), seem not to be associated with clinically relevant improvements in diabetic patients. Both approaches should carefully consider the context of application, patient expectations, their need for a long-term educational program and the need for adequate tools for data collection and sharing. Integration between healthcare providers should be improved to ensure patient-centered care and continuity of care. Further studies are needed to deepen the impact of the adherence to a PDTA program on patient clinical outcomes in the long term.

## Figures and Tables

**Table 1:** General characteristic of subjects included in the study. Data are expressed as median (I, III quartile) for continuous variables and absolute numbers (percentage) for categorical variables.

	*N*	PDTA (*N* = 91)	PDTA and CAD (*N* = 33)	All (*N* = 124)	*p*-Value
Sex	124				0.459
Male		54 (59%)	22 (67%)	76 (61%)	
Female		37 (41%)	11 (33%)	48 (39%)	
Age (years)	124	72.3 (66.5, 77.4)	69.6 (63.3, 75.0)	71.5 (66.1, 77.2)	0.080
Pharmacological treatment	124				0.052
Oral hypoglycemic		82 (90%)	29 (88%)	111 (90%)	
Insulin		1 (1%)	3 (9%)	4 (3%)	
None		8 (9%)	1 (3%)	9 (7%)	
Follow-up time (days)	123	367 (314, 403)	375 (348, 399)	370 (315, 403)	0.777

Abbreviations: PDTA, percorso diagnostico terapeutico assistenziale (diagnostic-therapeutic pathway program); CAD, Centro Anti-Diabetico; *N*, number.

**Table 2:** Descriptive table on biochemical data and clinical information collected during baseline PDTA examination. Data are expressed as median (I, III quartile) for continuous variables and absolute numbers (percentage) for categorical variables.

	*N*	PDTA (*N* = 91)	PDTA and CAD (*N* = 33)	All (*N* = 124)	*p*-Value
Glycated Hemoglobin (mmol/mol)	124	51.0 (45.5, 56.0)	57.0 (49.0, 74.0)	52.0 (46.0, 59.0)	0.018
Microalbuminuria (mg/die)	111	10.4 (3.3, 22.7)	6.5 (3.1, 15.0)	8.3 (3.1, 21.9)	0.192
Creatinine Clearance (mL/min)	120	86.7 (69.4, 103.3)	84.1 (66.5, 99.6)	85.7 (68.8, 102.8)	0.539
Total Cholesterol (mg/dL)	120	180 (158, 209)	176 (150, 222)	178 (152, 212)	0.840
LDL Cholesterol (mg/dL)	124	99.0 (77.0, 123.5)	89.0 (71.0, 139.0)	96.5 (76.0, 125.5)	0.902
HDL Cholesterol (mg/dL)	120	56.0 (44.0, 65.0)	49.0 (42.0, 54.0)	53.5 (43.0, 63.0)	0.039
Triglycerides (mg/dL)	118	102.0 (76.0,140.5)	119.0 (95.5, 178.5)	105.5 (78.2, 151.0)	0.043
Weight (kg)	124	80.0 (68.5, 89.0)	81.0 (75.0, 93.0)	80.0 (70.0, 90.0)	0.164
BMI (kg/m^2^)	124	28.5 (25.8, 30.8)	29.3 (25.8, 32.4)	28.7 (25.8, 31.2)	0.374
Waist Circumference (cm)	111	105 (96, 111)	107 (99, 114)	106 (97, 112)	0.524
Smoke habits	122				0.227
Current smokers		13 (15%)	3 (9%)	16 (13%)	
Non-smokers		50 (56%)	15 (45%)	65 (53%)	
Former smokers		26 (29%)	15 (45%)	41 (34%)	
Cigarettes per day (n/day)	57	20.0 (13.5, 27.5)	20.0 (20.0, 30.0)	20.0 (15.0, 30.0)	0.263
Alcohol consume (gr/day)	124	0 (0, 12)	0 (0, 12)	0 (0, 12)	0.159
Physical activity	124				0.579
None		29 (32%)	15 (45%)	44 (35%)	
Light physical activity		52 (57%)	15 (45%)	67 (54%)	
Moderate physical activity		7 (8%)	2 (6%)	9 (7%)	
Intense physical activity		3 (3%)	1 (3%)	4 (3%)	
Systolic Blood Pressure (mmHg)	124	150 (140, 160)	145 (135, 160)	148 (140, 160)	0.726
Diastolic Blood Pressure (mmHg)	124	80.0 (70.0, 87.0)	80.0 (75.0, 90.0)	80.0 (71.5, 90.0)	0.208
Heart Rate (bpm)	119	72.0 (66.0, 80.0)	75.5 (66.0, 80.0)	73.0 (66.0, 80.0)	0.752

Abbreviations: PDTA, percorso diagnostico terapeutico assistenziale (diagnostic-therapeutic pathway program); CAD, Centro Anti-Diabetico; *N*, number; LDL, low-density lipoprotein; HDL, high-density lipoprotein; BMI, body mass index.

**Table 3:** Descriptive table on biochemical data and clinical information collected during follow-up PDTA examination. Data are expressed as median (I, III quartile) for continuous variables and absolute numbers (percentage) for categorical variables.

	*N*	PDTA (*N* = 91)	PDTA and CAD (*N* = 33)	All (*N* = 124)	*p*-Value
Glycated Hemoglobin (mmol/mol)	123	50 (44, 55)	53 (49, 63)	51 (44, 57)	0.010
Microalbuminuria (mg/die)	107	10.6 (3.0, 32.5)	4.8 (2.3, 9.6)	7.9 (3.0, 27.2)	0.046
Creatinine Clearance (mL/min)	119	86.0 (74.1, 104.3)	78.4 (67.5, 99.2)	83.9 (71.8, 103.6)	0.182
Total Cholesterol (mg/dL)	121	173 (154, 199)	162 (151, 192)	172 (151, 199)	0.588
LDL Cholesterol (mg/dL)	120	94.0 (74.5, 118.5)	87.0 (72.0, 108.0)	91.5 (73.8, 118.0)	0.585
HDL Cholesterol (mg/dL)	120	54.0 (45.0, 67.0)	51.0 (41.0, 57.0)	52.5 (43.8, 64.5)	0.087
Triglycerides (mg/dL)	118	100 (74, 143)	120 (84, 163)	106 (77, 146)	0.212
Weight (kg)	124	79 (68, 87)	84 (74, 93)	80 (70, 90)	0.104
BMI (kg/m^2^)	124	28.0 (25.6, 31.0)	30.1 (25.5, 31.6)	28.4 (25.6, 31.4)	0.458
Waist Circumference (cm)	117	104 (96, 113)	107 (100, 114)	105 (96, 114)	0.322
Smoke habits	123				0.334
Current smokers		11 (12)	3 (9)	14 (11)	
Non-smokers		51 (57)	15 (45)	66 (54)	
Former smokers		28 (31)	15 (45)	43 (35)	
Cigarettes per day (n/day)	57	20.0 (11.5, 27.5)	20.0 (20.0, 30.0)	20.0 (15.0, 30.0)	0.164
Alcohol consume (gr/day)	124	0.0 (0.0, 14.4)	0.0 (0.0, 14.4)	0.0 (0.0, 14.4)	0.434
Physical activity	124				0.594
None		40 (44)	18 (55)	58 (47)	
Light physical activity		42 (46)	12 (36)	54 (44)	
Moderate physical activity		8 (9)	2 (6)	10 (8)	
Intense physical activity		1 (1)	1 (3)	2 (2)	
Systolic Blood Pressure (mmHg)	124	145 (135, 158)	140 (130, 160)	145 (130, 160)	0.320
Diastolic Blood Pressure (mmHg)	124	75 (70, 80)	80 (70, 90)	76 (70, 80)	0.235
Heart Rate (bpm)	124	73.0 (66.0, 84.0)	74.0 (68.0, 82.0)	73.0 (66.0, 83.2)	0.514

Abbreviations: PDTA, percorso diagnostico terapeutico assistenziale (diagnostic-therapeutic pathway program); CAD, Centro Anti-Diabetico; *N*, number; LDL, low-density lpoprotein; HDL, high-density lipoprotein; BMI, body mass index.

**Table 4:** Estimated change of biochemical and clinical variables from baseline to follow-up. Model 1 included only time as covariate. Model 2 included time and CAD enrollment as covariates and calculated the effects of variables separately (time effect and CAD effect), and in case of interaction (time × CAD effect).

	Model 1			Model 2								
				(Time Effect)			(CAD Effect)			(CAD × Time Effect)		
Variable	EE	SE	*p*-Value	EE	SE	*p*-Value	EE	SE	*p*-Value	EE	SE	*p*-Value
Glycated haemoglobin	−0.005	0.003	0.392	−0.005	0.003	0.392	8.230	2.063	0.001	−0.002	0.006	0.963
Microalbuminuria	0.003	0.010	0.934	0.003	0.010	0.934	−5.075	9.472	0.786	−0.036	0.022	0.963
Creatinine	0.009	0.009	0.658	0.009	0.009	0.658	−5.205	5.285	0.599	0.000	0.020	1
Total cholesterol	−0.020	0.009	0.159	−0.020	0.009	0.159	2.609	7.589	0.894	−0.015	0.021	0.963
Low density lipoprotein	−0.018	0.008	0.159	−0.018	0.008	0.159	3.619	6.600	0.786	−0.010	0.018	0.963
High density lipoprotein	0.003	0.002	0.623	0.003	0.002	0.623	−5.875	3.065	0.367	0.001	0.006	0.963
Triglycerides	0.001	0.012	0.934	0.001	0.012	0.934	16.389	11.206	0.504	−0.031	0.027	0.963
Weight	0.000	0.001	0.730	0.000	0.001	0.730	4.025	2.906	0.504	0.002	0.002	0.963
Body mass index	0.000	0.000	0.644	0.000	0.000	0.644	0.580	0.870	0.786	0.000	0.001	0.963
Waist circumference	0.000	0.001	0.735	0.000	0.001	0.735	2.496	2.325	0.599	0.000	0.002	0.966
Number of cigarettes	−0.001	0.000	0.161	−0.001	0.000	0.161	4.768	3.306	0.504	0.001	0.001	0.963
Alcohol	−0.009	0.002	<0.001	0.000	0.003	<0.001	0.590	2.784	0.894	0.000	0.001	0.963
Systolic blood pressure	−0.007	0.005	0.265	−0.007	0.005	0.265	−3.300	3.650	0.669	−0.010	0.012	0.963
Diastolic blood pressure	−0.011	0.003	0.001	−0.011	0.003	0.001	2.350	1.831	0.518	0.006	0.006	0.963
Heart rate	0.003	0.002	0.730	0.003	0.002	0.730	1.153	2.026	0.786	0.001	0.006	0.963
Phisical Activity (Moderate-Intense)	−0.001	0.001	0.309	−0.001	0.001	0.309	0.182	2.247	0.935	0.001	0.007	0.963
Smoker (Yes)	−0.002	0.003	0.481	−0.002	0.003	0.481	0.483	3.502	0.890	0.002	0.011	0.963

Abbreviations: CAD, Centro Anti-Diabetico; EE, estimated effect; SE, standard error.

## Data Availability

The data presented in this study are available upon request from the corresponding author.

## Supplementary Materials

The following are available online at https://www.mdpi.com/article/10.3390/ijerph182111540/s1, Figure S1: Flow diagram reporting the number of patients excluded at each stage of recruitment.
