# Supplementary material for: Evaluating Effectiveness of Outpatient Monitoring in Type 2 Diabetes: The One-Year Experience in an Italian Group of Primary Care
Source: Int J Environ Res Public Health. 2021 Nov 3;18(21):11540. doi: 10.3390/ijerph182111540 (PMC8583604; doi:10.3390/ijerph182111540)
Supplement: Supplementary file 1 [file ijerph-18-11540-s001.zip › ijerph-1384094-supplementary.pdf]

Figure S1. Flow diagram reporting the number of patients excluded at each stage of recruitment (based on STROBE Statement)

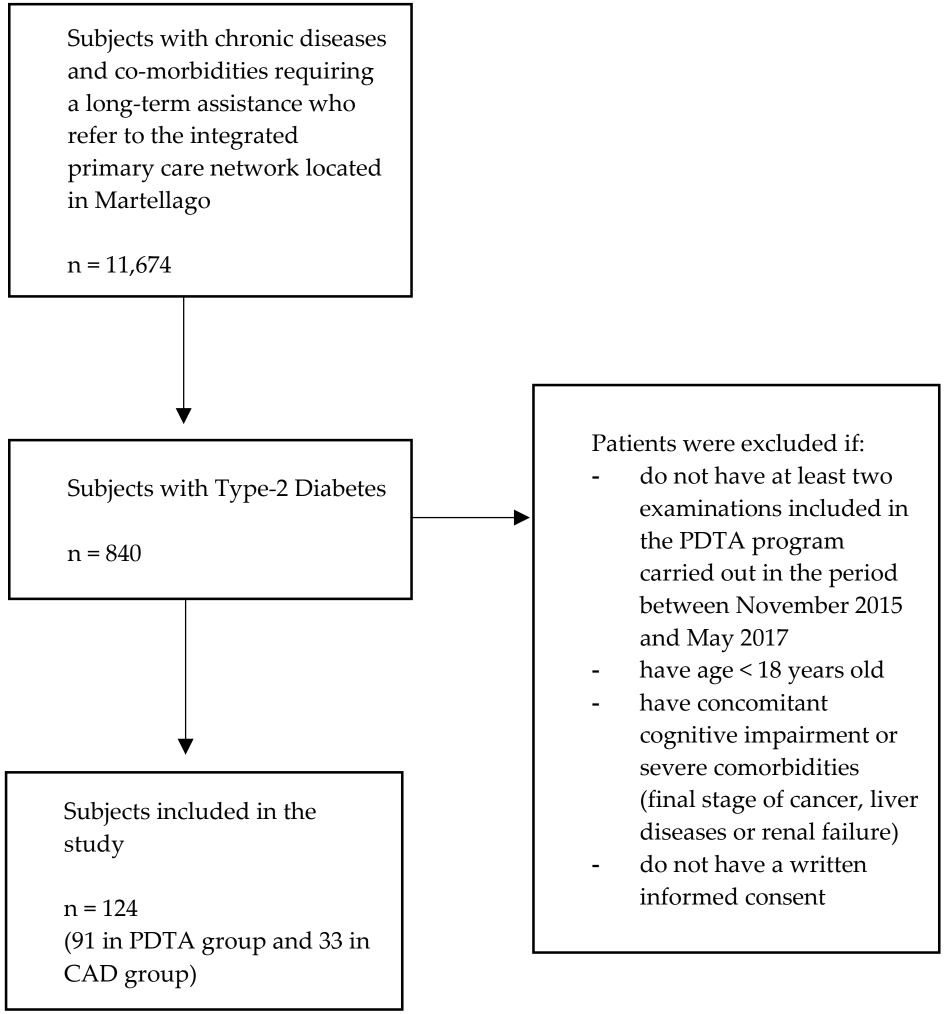

Von Elm E, Altman DG, Egger M, Pocock SJ, Gøtzsche PC, Vandenbroucke JP; STROBE Initiative. The Strengthening the Reporting of Observational Studies in Epidemiology (STROBE)statement: guidelines for reporting observational studies. J Clin Epidemiol. 2008 Apr;61(4):344-9. PMID: 18313558. https://www.strobe-statement.org/
